# Osteogenic potential of gingival stromal progenitor cells cultured in platelet rich fibrin is predicted by core-binding factor subunit-α1/Sox9 expression ratio ( *in vitro*)

**DOI:** 10.12688/f1000research.15423.1

**Published:** 2018-07-25

**Authors:** Alexander Patera Nugraha, Ida Bagus Narmada, Diah Savitri Ernawati, Aristika Dinaryanti, Eryk Hendrianto, Igo Syaiful Ihsan, Wibi Riawan, Fedik Abdul Rantam

**Affiliations:** 1 Graduate School of Immunology, Postgraduate School, Universitas Airlangga, Surabaya, 60132, Indonesia; 2 Orthodontic Department, Faculty of Dental Medicine, Universitas Airlangga, Surabaya, 60132, Indonesia; 3 Stem Cell Research and Development Center, Universitas Airlangga, Surabaya, 60132, Indonesia; 4 Oral Medicine Department, Faculty of Dental Medicine, Universitas Airlangga, Surabaya, 60132, Indonesia; 5 Biochemistry Biomolecular Laboratory, Faculty of Medicine, Universitas Brawijaya, Malang, 65145, Indonesia; 6 Virology and Immunology Laboratory, Microbiology Department, Faculty of Veterinary Medicine, Universitas Airlangga., Surabaya, 60132, Indonesia

**Keywords:** Core-Binding Factor Subunit-α, Gingival Stromal Progenitor Cells, Osteogenic Differentiation, Platelet Rich Fibrin, Sox9.

## Abstract

**Background: **Alveolar bone defect regeneration has long been problematic in the field of dentistry. Gingival stromal progenitor cells (GSPCs) offer a promising solution for alveolar bone regeneration. In order to optimally differentiate and proliferate progenitor cells, growth factors (GFs) are required. Platelet rich fibrin (PRF) has many GFs and can be easily manufactured. Core-binding factor subunit-α1 (CBF-α1) constitutes a well-known osteogenic differentiation transcription factor in SPCs. Sox9, as a chondrogenic transcription factor, interacts and inhibits CBF-α1, but its precise role in direct *in vitro *osteogenesis remains unknown. GSPCs cultured *in vitro* in PRF to optimally stimulate osteogenic differentiation has been largely overlooked. The aim of this study was to analyze GSPCs cultured in PRF osteogenic differentiation predicted by CBF-α1/Sox9.

**Methods**: This study used a true experimental with post-test only control group design and random sampling. GPSCs isolated from the lower gingiva of four healthy, 250-gram, 1-month old, male Wistar rats ( *Rattus Novergicus*) were cultured for two weeks, passaged every 4-5 days. GSPCs in passage 3-5 were cultured in five M24 plates (N=108; n=6/group) for Day 7, Day 14, and Day 21 in three different mediums (control negative group: αModified Eagle Medium; control positive group: High Glucose-Dulbecco’s Modified Eagle Medium (DMEM-HG) + osteogenic medium; Treatment group: DMEM-HG + osteogenic medium + PRF). CBF-α1 and Sox9 were examined with ICC monoclonal antibody. A one-way ANOVA continued with Tukey HSD test (p<0.05) based on Kolmogorov–Smirnov and Levene's tests (p>0.05) was performed.

**Results:** The treatment group showed the highest CBF-α1/Sox9 ratio (16.00±3.000/14.33±2.517) on Day 7, while the lowest CBF-α1/Sox9 ratio (3.33±1.528/3.67±1.155) occurred in the control negative group on Day 21, with significant difference between the groups (p<0.05).

**Conclusion:** GSPCs cultured in PRF had potential osteogenic differentiation ability predicted by the CBF-α1/sox9 ratio.

## Introduction

Dental caries represents a major global dental public health problem because of their high prevalence. The World Health Organization reported that almost 90% of people worldwide suffered from caries ^1^. Basic Health Research of National Health (RISKESDAS) in 2013 reported that 93,998,727 Indonesians, 53.2% of the population, suffered from active caries ^2^. Dental caries must be treated appropriately because, if neglected, they become so severe that the affected teeth must be extracted. Indeed, the most common cause of tooth loss is dental caries ^3^. Populations experiencing low socioeconomic conditions demonstrate higher prevalence and extent of tooth loss because of extremely limited access to dental treatment ^4^.

Tooth extraction has been the most common form of dental treatment performed in Indonesia that can lead to bone defects. RISKESDAS statistics dating from 2014 indicated that treatment involving tooth extraction reached as high as 79.6% of cases ^5^. A previous study of tooth extraction-related complications revealed the prevalence of fractures (31.82%), bleeding (4.54%) and swelling (2.27%) ^6^. Tooth extraction can lead to alveolar bone resorption and the destruction of alveolar bone components. Moreover, it may lead to resorption of the jawbone ^7^. Tooth extraction followed by buccolingual and apicocoronal dimension reduction of the alveolar ridge at the edentulous site might be performed due to bone defects ^8^.

Alveolar bone defect regeneration has long represented a challenge in the field of dentistry. Various efforts have been made to accelerate bone regeneration, such as using bone grafts. The most current treatment performed in relation to the alveolar bone involves the use of platelet rich fibrin (PRF). PRF materials encouraging bone regeneration therapy have significantly improved the clinical outcomes stemming from the treatment of infrabony defects. PRF has achieved this through the maintaining of space for tissue regeneration by inducing an osteoinductive and osteoconductive effect in the alveolar bone defect area ^9^.

Nowadays, alveolar bone defect treatment involving PRF using stromal progenitor cells (SPCs) is becoming increasingly widespread. SPCs have the advantage of being able to repair and regenerate various organs and tissue, and have been considerably used in bone tissue engineering, which offers encouraging solutions for bone regeneration ^10, 11^. SPCs are non-hematopoietic stromal cells. They have multipotent capabilities, including immunomodulators and immunoregulators, paracrine, autocrine action, and migrate directly to the tissue initiating healing and regeneration making SPCs particularly suitable for regenerative medicine development ^12– 14^.

The orofacial region is a unique and rich source of SPCs. Those contained in the oral cavity and tooth tissue represent an emerging interesting and topical object for investigation because isolating progenitor cells from the oral tissues can be achieved with minimal invasive procedures compared to bone marrow mesenchymal stem cell (BMSC) obtainment. The utilization of progenitor cells from the oral cavity is still rarely studied and applied. However, it is potentially advantageous for tissue regeneration and, therefore, merits further investigation ^15^.

The SPCs that are potentially useful as part of regenerative alveolar bone therapy are gingival stromal progenitor cells (GSPCs) derived from hyperplastic gingival tissue (gum overgrowth) by means of a gingivectomy. GSPCs have phenotypic characteristics and abilities similar to those of BMSCs. GSPCs possess self-renewal capabilities and also demonstrate the specific ability to regenerate into alveolar bone when transplanted into immunocompromised mice. GSPCs also specifically induce bone matrix formation in lamellar structures by recruiting host cells ^11, 15– 17^. The osteogenic ability of GSPCs needs to be explored for further application and therapy.

During skeletal formation, master transcription genes such core-binding factor subunit-α (CBF-α1) and Osterix have been identified ^18^. However, their specific and distinct roles in various tissue types are still unclear. Sox9 is well known as a master gene regulator during chondrogenic differentiation, while CBF-α1 plays an important role during osteogenic differentiation. GSPCs, as osteoprogenitors and chondroprogenitors, express Sox9 and Runx2 during skeletal formation condensation. There is also a direct interaction between Sox9 and CBF-α1, which inhibits Sox9 activity ^18^. Sox9 inhibitory effect on osteoblast maturation through CBF-α1 is an essential mechanism for osteo-chondroprogenitor fate determination ^19^.

In order for GSPCs to differentiate and proliferate optimally they require growth factors (GFs), various varieties of which are shown to promote osteogenic differentiation of SPCs *in vitro*. PRF is predicted to be combined to promote SPCs osteogenic differentiation and ensure mineralization *in vitro* ^19^. PRF can be easily produced by centrifuging without anticoagulants. PRF is rich in GFs consisting of platelet derived growth factor-β (PDGF-β), transforming growth factor-β1 (TGFβ-1), vascular endothelial growth factor (VEGF) and insulin growth factor (IGF-I). PRF provides an effective scaffold to facilitate osteogenic differentiation of GSPCs ^20– 23^.

The osteogenic differentiation of GSPCs can be detected by various osteogenic marker expressions, such as CBF-α1 expression. The observed osteogenic markers of GSPCs are CBF subunit-α1 (CBF-α1) and Sox9 ^24, 25^. Nonetheless, there is insufficient information regarding Sox9’s role in osteogenesis of GPSCs *in vitro*. A study conducted by Stockl *et al.* mentioned that Sox9 plays a positive proliferative role in inhibiting and delaying osteogenic differentiation in rat SPCs ^26^.

The hypothesis of the current study is that GSPCs cultured in PRF can increase the CBF-α1/Sox9 expression ratio during osteogenic differentiation. Furthermore, a second objective was to analyze GSPCs cultured in PRF osteogenic differentiation predicted by CBF-α1/Sox9 expression ratio.

## Methods

### Ethical clearance

This study received ethical clearance relating to animal subjects from the Ethics Research Committee, Faculty of Dental Medicine, Universitas Airlangga, Surabaya, East Java, Indonesia (number 289/HRECC.FODM/XII/2017). The research was conducted at an experimental laboratory within the Stem Cell and Tissue Engineering Development Centre, Universitas Airlangga.

### Research design and experimental animals

The research was fully experimental with a post-test only control group design. Sample groups were selected by means of simple random number sampling. Each animal was assigned a unique number, which were picked out of a hat by a blindfolded researcher.

The subjects consisted of male Wistar rats ( *Rattus norvegicus*; n=4), who were adapted to the environment for 7 days. Wistar rats were obtained and cared for at the Stem Cell Animal Laboratory, Universitas Airlangga. All animals were housed in polycarbonate cages, subjected to a 12-hour light-dark cycle at the constant temperature of 23°C, and fed a standard pellet diet (expanded pellets; Stepfield, UK) with tap water *ad libitum* at a temperature of 22°C±2°C.

GPSCs were isolated from the lower gingival tissue of four 1-month old, healthy, mean weight = 250g, male rats through a gingivectomy, before the rats were euthanized with doses 60mg/body weight of ketamine and xylazine. Animal suffering was reduced when removing the GPSCs using rodent’s anesthesia (intramuscular injection at 0.05–0.1ml/10g body weight rodent anesthesia: ketamine, xylazine, acepromazine, and sterile isotonic saline; Sigma Aldrich, USA) following Duan *et al’s* method ^23^.

GPSCs was passaged every 4–5 days following Rantam *et al*’s SPCs culture method ^27^. GSPCs in passage 3–5 were cultured in five M24 plates (Sigma-Aldrich) (N=108; n=6/group) until Day 7, Day 14 and Day 21 in three different culture mediums (control negative group, control positive group and treatment group; see below for details).

Sample size (n=4 for GPSCs isolation; n=36 for PRF isolation) was based on Lemeshow's formula to determine minimum sample size

### Platelet rich fibrin isolation

A different population of rats were used for PRF isolation (n=36; 36 month old; mean weight = 250g). These male Wistar rats were maintained as above. Blood was aspirated through the left ventricle of each animals’ heart, after anesthesia had been administered by injection using a 60mg/body weight dose of ketamine and a 3mg/body weight dose of xylazine (Sigma Aldrich). 1.5ml of blood was aspirated using a 3ml disposable syringe and then inserted in a vacutainer tube without an anticoagulant before being centrifuged at 3000 rpm/min for 10min (Kubota, Tokyo, Japan). The centrifuging was performed by inserting two balance tubes containing water with the same weight as the tube of blood. When the tube is removed from the centrifuge, three layers will appear that are divided into three sections; the lower section consists of red blood cells, the middle section contains PRF and the upper section is formed of acellular plasma. The PRF was then isolated after which the PRF was cut into small pieces using sterile scissors and inserted into each culture plate of the treatment group ^22, 28, 29^.

### Osteogenic differentiation in a combination of platelet rich fibrin and gingival stromal progenitor cells

The analysis was conducted on three groups, consisting of two control groups and one experimental group.

*GSPC treatment group:* GSPCs were cultured with PRF and containing ITS plus, 2mM L-glutamine, 100μg/ml sodium pyruvate, 0.2mM ascorbic acid-2 phosphate, dexamethasone 10-7 M (GeneTex, Taiwan), 10ng/ml TGF-β3 and high-dose glucose-Dulbecco's Modified Eagle Medium (DMEM-HG) (Sigma Aldrich).

*Positive control group:* GSPCs were placed on an osteogenic medium culture plate of ITS plus, 2 mM L-glutamine, 100μg/ml sodium pyruvate 0.2mM ascorbic acid-2 phosphate, dexamethasone 10-7 M (GeneTex).

*Negative control group:* GSPCs were cultured with αModified Eagle Medium (αMEM) (Sigma Aldrich).

Every three days, every group cell medium was replaced. Osteogenic differentiation was evaluated on Day 7, 14, 21 culture cells groups ^16^.

GSPCs cultured cells were coated with coverslips and, after incubation at 37°C for 1 - 2 hours, were fixed using 10% formaldehyde for 15 min. The coverslips were then rinsed four times with PBS and dried for several minutes. The cells were blocked with PBS and FBS 1% for 15–30 minutes and washed with PBS four times. The samples were then examined following immunocytochemical staining by indirect technique using a 3.3'-diaminobenzidine stain kit (Pierce DAB Substrate Paint Kit 34002, Thermofisher ^TM^, Waltham, MA, USA) and monoclonal antibodies (Santa Cruz Biotechnology, Dallas, TX, USA): anti-CBF-α1 (mouse monoclonal; sc-101145) and anti-Sox9 (mouse monoclonal; sc-166505). CBF-α1 and Sox9 expression was read using a light microscope (CX22 Binocular, Olympus) at 200x magnification. Every cell expressing CBF1-α or Sox9 in one field was examined three times by three experts (WR, EH and FAR) and the mean was then calculated ^27, 30, 31^.

### Data analysis

The data obtained was analyzed using ANOVA continued with Tukey HSD test (p<0.05) based on a Saphiro-Wilk normality test and a Levene's variance of homogeneity test (p>0.05). Data were analyzed using SPSS version 20.0 (IBM SPSS, Chicago, USA).

The experiments were replicated 3 times (n=54). The data was then duplicated (n=108) using an estimation formula and SPSS (see Supplementary File 1 and Supplementary File 2) ^32^.

## Results

**Figure 1.**
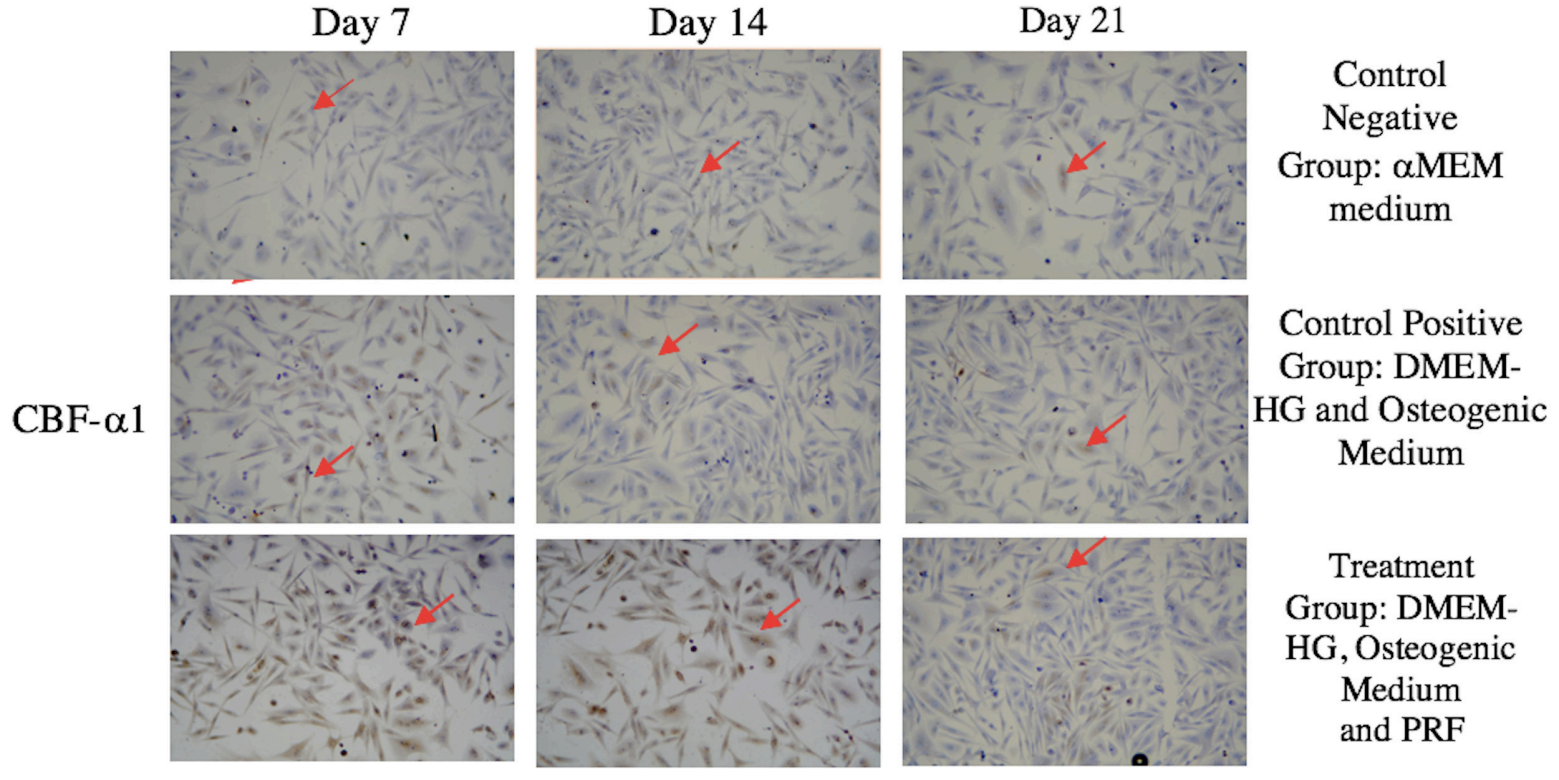
Core-binding factor subunit-α1 (CBF-α1) expression in gingival stromal progenitor cells (GSPCs) of Wistar rats ( *Rattus Novergicus*). (A-C) CBF-α1 expression in the negative control group; (D-F) CBF-α1 expression in the positive control group; (G-I) CBF-α1 expression in the treatment group. CBF-α1 expression in GSPCs was observed on Days 7, 14 and 21. Positive CBF-α1 expression is highlighted in brown (red arrow) following an examination at 200x magnification (n=1).

**Figure 2.**
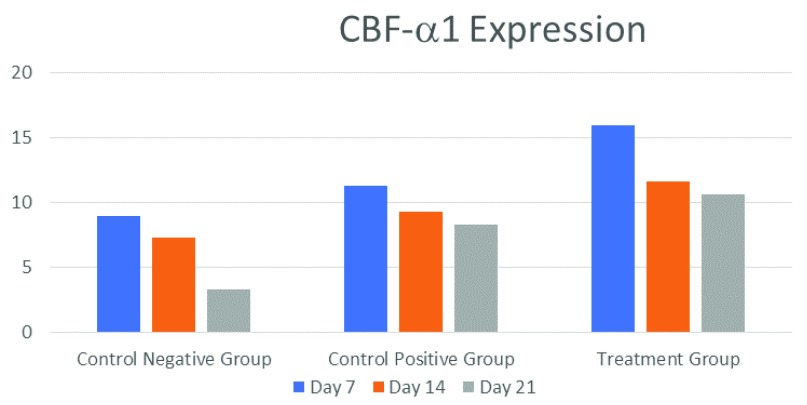
Mean core-binding factor subunit-α1 (CBF-α1) expression on Days 7, 14, 21 in each treatment group (n=6).

**Figure 3.**
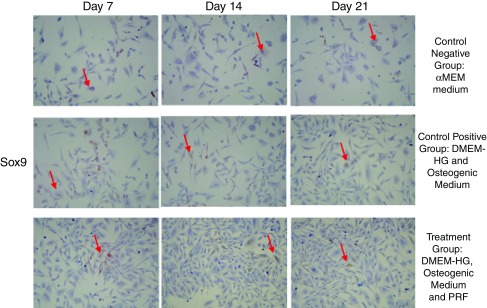
Sox9 expression in gingival stromal progenitor cells (GSPCs) of Wistar rats ( *Rattus Novergicus*). (A-C) Sox9 expression in the negative control group; (D-F) Sox9 expression in the positive control group; (G-I) Sox9 expression in the treatment group. Sox9 expression in GSPCs was observed on Days 7, 14 and 21. Positive Sox9 expression is highlighted in brown (red arrow) following examination at 200x magnification (n=1).

**Figure 4.**
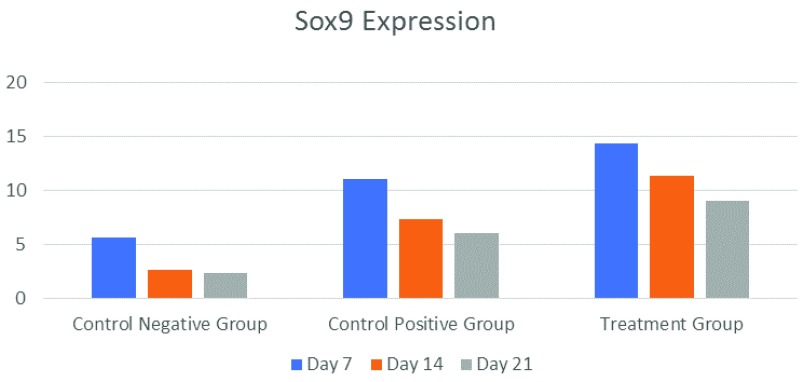
Mean Sox9 expression on Days 7, 14, 21 in each treatment group (n=6).

The highest average CBF-α1 expression was in the treatment group on Day 7, whereas the lowest was in the control (-) group on Day 21 ( Figure 1 and Figure 2). Sox9 expression had the highest mean value in the treatment group on Day 7, while its lowest value was in the negative control group on Day 21 ( Figure 3 and Figure 4).

The treatment group recorded the highest CBF-α1/Sox9 ratio (16.00±3.000/14.33±2.517/) on Day 7 while the lowest CBF-α1/Sox9 ratio (3.33±1.528/3.67±1.155) was registered by the control negative group on Day 21 ( Table 1). The data obtained was normal with homogeneous distribution (p>0.05). There was significant difference between CBF-α1 and Sox9 expression in each group (p<0.05) ( Supplementary Table 1 and Supplementary Table 2).

**Table 1.** CBF-α1/Sox9 expression ratio between groups.

Day	CBF-α1 expression			Sox9 expression			P-value *
	Negative control group	Positive control group	Treatment group	Negative control group	Positive control group	Treatment group	
7	9.00±2.000	11.33±1.528	16.00±3.000	5.67±1.155	11.00±1.00	14.33±2.517	0.00
14	7.33±1.528	9.33±0.577	11.67±2.082	2.67±0.577	7.33±1.528	11.33±1.528	0.00
21	3.33±1.528	8.33±1.155	10.67±1.528	2.33±1.155	6.00±1.000	9.00±2.000	0.00

Results are presented as the mean ± standard deviation. *One-way ANOVA, significant at p<0.05. CBF-α1: core-binding factor subunit-α1.

Raw results for CBF-α1 and Sox9 expression for all time points for all treatment groups (N=108; n=6/group) Click here for additional data file. Copyright: © 2018 Nugraha AP et al. 2018 Data associated with the article are available under the terms of the Creative Commons Zero "No rights reserved" data waiver (CC0 1.0 Public domain dedication).

Raw image data Click here for additional data file. Copyright: © 2018 Nugraha AP et al. 2018 Data associated with the article are available under the terms of the Creative Commons Zero "No rights reserved" data waiver (CC0 1.0 Public domain dedication).

## Discussion

GSPCs cultured in PRF expressed CBF-α1 strongly. In this study, the highest CBF-α1 expression was recorded by the treatment group on Day 7, with significant difference between groups. The CBF-α1 expression declined between Day 14 and Day 21. The results of this study were in line with the research by Zou *et al*., which suggested that CBF-α1 expression is used to detect the osteogenic ability of SPCs using yellow fluorescent protein ^33^.

CBF-α1 is a master key gene transcription factor associated with osteoblast differentiation, which initiates temporally and spatially controlled osteogenesis. Disturbances to CBF-α1 result in obstacles to bone formation because osteoblast differentiation cannot occur. Loss of CBF-α1 expression gene function in the early stages will interfere with osteogenic differentiation and homeostasis in bone development. CBF-α1 is often expressed strongly between Day 7 and Day 14 ^23, 25^. Osterix and CBF-α1 periodically regulate osteoblast differentiation processes ^34, 35^. A study conducted by Loebel *et al*. showed that CBF-α1 expression increased on Day 7, while Duan *et al*.’s study demonstrated that CBF-α1 expression increased on Day 12 as detected by RT-PCR ^19, 23^. Such findings differed from the results of this study due to the contrasting methods and samples employed, but there were similarities in that CBF-α1 was an early marker of osteogenic differentiation.

CBF-α1 plays an important role in the early stages of BMSCs differentiation into preosteoblasts. CBF-α1 is generally a preliminary regulator and Osterix is a regulator activator during osteoblast differentiation. Both of these osteoblastogenic coding genes are stimulated and regulated by various signaling pathways, such as the canonical Wnt signaling pathway and bone morphogenetic protein (BMP). Wnt/Cytosolic β-catenin stimulates osteoblastogenesis through the activation of osteogenic transcription factors CBF-α1 and Osterix ^36^. CBF-α1 is known as an important regulatory gene during osteogenic development by enhancing specific osteoblastic differentiation by inducing osteogenic extracellular matrix gene expression during osteoblast maturation, such as collagen-Iα, alkaline phosphatase, and osteocalcin ^33^.

In the present study, the GSPCs cultured in PRF stimulates CBF-α1 expression because PRF is rich in various GFs, such as TGFβ-1, PDGF, IGF, VEGF, FGF, EGF, and HGF. PRF promotes migration, proliferation and differentiation of mesenchmymal stem cells as well as neovascularization and collagen synthesis. PRF also promotes, accelerates and improves the quality of soft and bone tissue regeneration ^23^. According to Li *et al*., PRF significantly promotes the induction of mineralization of progenitor cells in alveolar bone, and endogenous stem cells present in the dental tissue that increases exclusively in CBF-α1 expression ^37, 38^.

Interestingly, GSPCs cultured PRF in this study increased Sox9 even in an osteogenic culture medium with significant difference with the control groups. In this study, the highest Sox9 expression occurred in the treatment group on Day 7. The results of this study were supported by those of a study by Sumarta *et al.,* which stated that SPCs cultured in PRF stimulate Sox9 expression ^22^. Sox9 expression showed a positive expression, thereby establishing the role of Sox9 during bone formation. In a knockout Sox9 animal model, osteogenic differentiation was also delayed ^39^. Significantly, recent genetics studies stated that Sox9 in SPCs could eventually differentiate into osteoblasts ^40^. Therefore, the inhibitory effect of Sox9 on osteoblastic and chondrocyte maturation via repression of CBF-alpha1 function is an essential mechanism for osteo-chondroprogenitor cell fate determination ^41^.

In this study, GSPCs-cultured PRF regulated and stimulated both CBF-α1/Sox9 expression ratio on Day 7 with significant difference between groups. The interaction and cooperation between CBF-α1/Sox9 is a mandatory master transcription gene for cartilage and bone development ^18^. As Sox9 inhibited and downregulated CBF-α1 on Day 7, it may be even more sensitive to predict osteogenic differentiation ability of SPCs. Furthermore, while Sox9 expression was downregulated, osteogenic differentiation ability was stimulated during early osteogenic differentiation *in vitro*. Nevertheless, CBF-α1/Sox9 expression ratio on Day 7 could be used to predict the osteogenic differentiation ability of GMSCs, suggesting a balance between CBF-α1/Sox9 in the earlier regulatory bone formation and regeneration ^19, 41^.

## Conclusion

GSPCs cultured in PRF increased CBF-α1/Sox9 expression on Day 7. GSPCs cultured in PRF possessed potential osteogenic differentiation ability as predicted by the CBF-α1/sox9 expression ratio. CBF-α1/Sox9 expression constitutes a promising future *in vitro* screening method employed to detect the earliest osteogenic differentiation of SPCs. Further study is required to analyze any association with CBF-α1/Sox9 expression ratio *in vivo.*

## Data availability

The data referenced by this article are under copyright with the following copyright statement: Copyright: © 2018 Nugraha AP et al.

Data associated with the article are available under the terms of the Creative Commons Zero "No rights reserved" data waiver (CC0 1.0 Public domain dedication). 

Dataset 1: Raw results for CBF-α1 and Sox9 expression for all time points for all treatment groups (N=108; n=6/group). DOI, 10.5256/f1000research.15423.d210638 ^42^

Dataset 2: Raw image data. DOI, 10.5256/f1000research.15423.d210639 ^43^

## Supplementary material

Supplementary Table 1. Tukey HSD multiple comparison between groups of CBF-α1.

Supplementary Table 2. Tukey HSD multiple comparison between groups of Sox9 expression.

Supplementary File 1: Estimation resume of CBF-α1 expression.

Supplementary File 2: Estimation resume of Sox9 expression.
