# Supplementary material for: Osteogenic potential of gingival stromal progenitor cells cultured in platelet rich fibrin is predicted by core-binding factor subunit-α1/Sox9 expression ratio ( in vitro)
Source: F1000Res. 2018 Jul 25;7:1134. [Version 1] doi: 10.12688/f1000research.15423.1 (PMC6097418; doi:10.12688/f1000research.15423.1)
Supplement: Supplementary file 3 [file f1000research-7-16808-s0002.tgz › 62700dc4-244b-4201-ad46-db4f3c3836f8.docx]

**Supplementary Table 1. Tukey HSD multiple comparison between groups of CBF-α1.**

| Group | | Groups | P value* |
| --- | --- | --- | --- |
| CBF-α1 | Control negative Day 7 | Control Negative Day 14 (CBF-α1) | 0.452 |
|  |  | Control Negative Day 21 (CBF-α1) | 0.000 |
|  |  | Control Positive Day 7 (CBF-α1) | 0.092 |
|  |  | Control Positive Day 14 (CBF-α1) | 0.998 |
|  |  | Control Positive Day 21 (CBF-α1) | 0.998 |
|  |  | Treatment Day 7 (CBF-α1) | 0.000 |
|  |  | Treatment Day 14 (CBF-α1) | 0.002 |
|  |  | Treatment Day 21 (CBF-α1) | 0.327 |
|  | Control Negative Day 14 | Control Negative Day 7 (CBF-α1) | 0.452 |
|  |  | Control Negative Day 21 (CBF-α1) | 0.000 |
|  |  | Control Positive Day 7 (CBF-α1) | 0.000 |
|  |  | Control Positive Day 14 (CBF-α1) | 0.125 |
|  |  | Control Positive Day 21 (CBF-α1) | 0.886 |
|  |  | Treatment Day 7 (CBF-α1) | 0.000 |
|  |  | Treatment Day 14 (CBF-α1) | 0.000 |
|  |  | Treatment Day 21 (CBF-α1) | 0.001 |
|  | Control Negative Day 21 | Control Negative Day 7 (CBF-α1) | 0.000 |
|  |  | Control Negative Day 14 (CBF-α1) | 0.000 |
|  |  | Control Positive Day 7 (CBF-α1) | 0.000 |
|  |  | Control Positive Day 14 (CBF-α1) | 0.000 |
|  |  | Control Positive Day 21 (CBF-α1) | 0.000 |
|  |  | Treatment Day 7 (CBF-α1) | 0.000 |
|  |  | Treatment Day 14 (CBF-α1) | 0.000 |
|  |  | Treatment Day 21 (CBF-α1) | 0.000 |
|  | Control Positive Day 7 | Control Negative Day 7 (CBF-α1) | 0.092 |
|  |  | Control Negative Day 14 (CBF-α1) | 0.000 |
|  |  | Control Negative Day 21 (CBF-α1) | 0.000 |
|  |  | Control Positive Day 14 (CBF-α1) | 0.369 |
|  |  | Control Positive Day 21 (CBF-α1) | 0.014 |
|  |  | Treatment Day 7 (CBF-α1) | 0.000 |
|  |  | Treatment Day 14 (CBF-α1) | 0.879 |
|  |  | Treatment Day 21 (CBF-α1) | 0.999 |
|  | Control Positive Day 14 | Control Negative Day 7 (CBF-α1) | 0.998 |
|  |  | Control Negative Day 14 (CBF-α1) | 0.125 |
|  |  | Control Negative Day 21 (CBF-α1) | 0.000 |
|  |  | Control Positive Day 7 (CBF-α1) | 0.369 |
|  |  | Control Positive Day 21 (CBF-α1) | 0.871 |
|  |  | Treatment Day 7 (CBF-α1) | 0.000 |
|  |  | Treatment Day 14 (CBF-α1) | 0.015 |
|  |  | Treatment Day 21 (CBF-α1) | 0.771 |
|  | Control Positive Day 21 | Control Negative Day 7 (CBF-α1) | 0.998 |
|  |  | Control Negative Day 14 (CBF-α1) | 0.886 |
|  |  | Control Negative Day 21 (CBF-α1) | 0.000 |
|  |  | Control Positive Day 7 (CBF-α1) | 0.014 |
|  |  | Control Positive Day 14 (CBF-α1) | 0.871 |
|  |  | Treatment Day 7 (CBF-α1) | 0.000 |
|  |  | Treatment Day 14 (CBF-α1) | 0.000 |
|  |  | Treatment Day 21 (CBF-α1) | 0.073 |
|  | Treatment Day 7 | Control Negative Day 7 (CBF-α1) | 0.000 |
|  |  | Control Negative Day 14 (CBF-α1) | 0.000 |
|  |  | Control Negative Day 21 (CBF-α1) | 0.000 |
|  |  | Control Positive Day 7 (CBF-α1) | 0.000 |
|  |  | Control Positive Day 14 (CBF-α1) | 0.000 |
|  |  | Control Positive Day 21 (CBF-α1) | 0.000 |
|  |  | Treatment Day 14 (CBF-α1) | 0.002 |
|  |  | Treatment Day 21 (CBF-α1) | 0.000 |
|  | Treatment Day 14 | Control Negative Day 7 (CBF-α1) | 0.002 |
|  |  | Control Negative Day 14 (CBF-α1) | 0.000 |
|  |  | Control Negative Day 21 (CBF-α1) | 0.000 |
|  |  | Control Positive Day 7 (CBF-α1) | 0.879 |
|  |  | Control Positive Day 14 (CBF-α1) | 0.015 |
|  |  | Control Positive Day 21 (CBF-α1) | 0.000 |
|  |  | Treatment Day 7 (CBF-α1) | 0.002 |
|  |  | Treatment Day 21 (CBF-α1) | 0.505 |
|  | Treatment Day 21 | Control Negative Day 7 (CBF-α1) | 0.327 |
|  |  | Control Negative Day 14 (CBF-α1) | 0.001 |
|  |  | Control Negative Day 21 (CBF-α1) | 0.000 |
|  |  | Control Positive Day 7 (CBF-α1) | 0.999 |
|  |  | Control Positive Day 14 (CBF-α1) | 0.771 |
|  |  | Control Positive Day 21 (CBF-α1) | 0.073 |
|  |  | Treatment Day 7 (CBF-α1) | 0.000 |
|  |  | Treatment Day 14 (CBF-α1) | 0.505 |

CBF-α1, core-binding factor subunit-α.

*Significant at p<0.05.
