# Supplementary material for: Osteogenic potential of gingival stromal progenitor cells cultured in platelet rich fibrin is predicted by core-binding factor subunit-α1/Sox9 expression ratio ( in vitro)
Source: F1000Res. 2018 Jul 25;7:1134. [Version 1] doi: 10.12688/f1000research.15423.1 (PMC6097418; doi:10.12688/f1000research.15423.1)
Supplement: Supplementary file 4 [file f1000research-7-16808-s0003.tgz › 2501cf1f-74ef-44f1-91c8-120a7abd6cd0.docx]

**Supplementary Table 2. Tukey HSD multiple comparison between groups of Sox9 expression.**

|  | | | | | | |
| --- | --- | --- | --- | --- | --- | --- |
| (I) Group | (J) Group | Mean Difference (I-J) | Std. Error | Sig. | 95% Confidence Interval | |
|  |  |  |  |  | Lower Bound | Upper Bound |
| Control Negative Day 7 (SOX9) | Control Negative Day 14 (SOX9) | 3,21167^*^ | ,62406 | ,000 | 1,1790 | 5,2443 |
|  | Control Negative Day 21 (SOX9) | 3,72667^*^ | ,62406 | ,000 | 1,6940 | 5,7593 |
|  | Control Positive Day 7 (SOX9) | -5,37333^*^ | ,62406 | ,000 | -7,4060 | -3,3407 |
|  | Control Positive Day 14 (SOX9) | -1,26000 | ,62406 | ,539 | -3,2926 | ,7726 |
|  | Control Positive Day 21 (SOX9) | -,04667 | ,62406 | 1,000 | -2,0793 | 1,9860 |
|  | Treatment Day 7 (SOX9) | -8,37667^*^ | ,62406 | ,000 | -10,4093 | -6,3440 |
|  | Treatment Day 14 (SOX9) | -5,27500^*^ | ,62406 | ,000 | -7,3076 | -3,2424 |
|  | Treatment Day 21 (SOX9 | -3,34000^*^ | ,62406 | ,000 | -5,3726 | -1,3074 |
| Control Negative Day 14 (SOX9) | Control Negative Day 7 (SOX9) | -3,21167^*^ | ,62406 | ,000 | -5,2443 | -1,1790 |
|  | Control Negative Day 21 (SOX9) | ,51500 | ,62406 | ,995 | -1,5176 | 2,5476 |
|  | Control Positive Day 7 (SOX9) | -8,58500^*^ | ,62406 | ,000 | -10,6176 | -6,5524 |
|  | Control Positive Day 14 (SOX9) | -4,47167^*^ | ,62406 | ,000 | -6,5043 | -2,4390 |
|  | Control Positive Day 21 (SOX9) | -3,25833^*^ | ,62406 | ,000 | -5,2910 | -1,2257 |
|  | Treatment Day 7 (SOX9) | -11,58833^*^ | ,62406 | ,000 | -13,6210 | -9,5557 |
|  | Treatment Day 14 (SOX9) | -8,48667^*^ | ,62406 | ,000 | -10,5193 | -6,4540 |
|  | Treatment Day 21 (SOX9 | -6,55167^*^ | ,62406 | ,000 | -8,5843 | -4,5190 |
| Control Negative Day 21 (SOX9) | Control Negative Day 7 (SOX9) | -3,72667^*^ | ,62406 | ,000 | -5,7593 | -1,6940 |
|  | Control Negative Day 14 (SOX9) | -,51500 | ,62406 | ,995 | -2,5476 | 1,5176 |
|  | Control Positive Day 7 (SOX9) | -9,10000^*^ | ,62406 | ,000 | -11,1326 | -7,0674 |
|  | Control Positive Day 14 (SOX9) | -4,98667^*^ | ,62406 | ,000 | -7,0193 | -2,9540 |
|  | Control Positive Day 21 (SOX9) | -3,77333^*^ | ,62406 | ,000 | -5,8060 | -1,7407 |
|  | Treatment Day 7 (SOX9) | -12,10333^*^ | ,62406 | ,000 | -14,1360 | -10,0707 |
|  | Treatment Day 14 (SOX9) | -9,00167^*^ | ,62406 | ,000 | -11,0343 | -6,9690 |
|  | Treatment Day 21 (SOX9 | -7,06667^*^ | ,62406 | ,000 | -9,0993 | -5,0340 |
| Control Positive Day 7 (SOX9) | Control Negative Day 7 (SOX9) | 5,37333^*^ | ,62406 | ,000 | 3,3407 | 7,4060 |
|  | Control Negative Day 14 (SOX9) | 8,58500^*^ | ,62406 | ,000 | 6,5524 | 10,6176 |
|  | Control Negative Day 21 (SOX9) | 9,10000^*^ | ,62406 | ,000 | 7,0674 | 11,1326 |
|  | Control Positive Day 14 (SOX9) | 4,11333^*^ | ,62406 | ,000 | 2,0807 | 6,1460 |
|  | Control Positive Day 21 (SOX9) | 5,32667^*^ | ,62406 | ,000 | 3,2940 | 7,3593 |
|  | Treatment Day 7 (SOX9) | -3,00333^*^ | ,62406 | ,001 | -5,0360 | -,9707 |
|  | Treatment Day 14 (SOX9) | ,09833 | ,62406 | 1,000 | -1,9343 | 2,1310 |
|  | Treatment Day 21 (SOX9 | 2,03333^*^ | ,62406 | ,050 | ,0007 | 4,0660 |
| Control Positive Day 14 (SOX9) | Control Negative Day 7 (SOX9) | 1,26000 | ,62406 | ,539 | -,7726 | 3,2926 |
|  | Control Negative Day 14 (SOX9) | 4,47167^*^ | ,62406 | ,000 | 2,4390 | 6,5043 |
|  | Control Negative Day 21 (SOX9) | 4,98667^*^ | ,62406 | ,000 | 2,9540 | 7,0193 |
|  | Control Positive Day 7 (SOX9) | -4,11333^*^ | ,62406 | ,000 | -6,1460 | -2,0807 |
|  | Control Positive Day 21 (SOX9) | 1,21333 | ,62406 | ,588 | -,8193 | 3,2460 |
|  | Treatment Day 7 (SOX9) | -7,11667^*^ | ,62406 | ,000 | -9,1493 | -5,0840 |
|  | Treatment Day 14 (SOX9) | -4,01500^*^ | ,62406 | ,000 | -6,0476 | -1,9824 |
|  | Treatment Day 21 (SOX9 | -2,08000^*^ | ,62406 | ,041 | -4,1126 | -,0474 |
| Control Positive Day 21 (SOX9) | Control Negative Day 7 (SOX9) | ,04667 | ,62406 | 1,000 | -1,9860 | 2,0793 |
|  | Control Negative Day 14 (SOX9) | 3,25833^*^ | ,62406 | ,000 | 1,2257 | 5,2910 |
|  | Control Negative Day 21 (SOX9) | 3,77333^*^ | ,62406 | ,000 | 1,7407 | 5,8060 |
|  | Control Positive Day 7 (SOX9) | -5,32667^*^ | ,62406 | ,000 | -7,3593 | -3,2940 |
|  | Control Positive Day 14 (SOX9) | -1,21333 | ,62406 | ,588 | -3,2460 | ,8193 |
|  | Treatment Day 7 (SOX9) | -8,33000^*^ | ,62406 | ,000 | -10,3626 | -6,2974 |
|  | Treatment Day 14 (SOX9) | -5,22833^*^ | ,62406 | ,000 | -7,2610 | -3,1957 |
|  | Treatment Day 21 (SOX9 | -3,29333^*^ | ,62406 | ,000 | -5,3260 | -1,2607 |
| Treatment Day 7 (SOX9) | Control Negative Day 7 (SOX9) | 8,37667^*^ | ,62406 | ,000 | 6,3440 | 10,4093 |
|  | Control Negative Day 14 (SOX9) | 11,58833^*^ | ,62406 | ,000 | 9,5557 | 13,6210 |
|  | Control Negative Day 21 (SOX9) | 12,10333^*^ | ,62406 | ,000 | 10,0707 | 14,1360 |
|  | Control Positive Day 7 (SOX9) | 3,00333^*^ | ,62406 | ,001 | ,9707 | 5,0360 |
|  | Control Positive Day 14 (SOX9) | 7,11667^*^ | ,62406 | ,000 | 5,0840 | 9,1493 |
|  | Control Positive Day 21 (SOX9) | 8,33000^*^ | ,62406 | ,000 | 6,2974 | 10,3626 |
|  | Treatment Day 14 (SOX9) | 3,10167^*^ | ,62406 | ,000 | 1,0690 | 5,1343 |
|  | Treatment Day 21 (SOX9 | 5,03667^*^ | ,62406 | ,000 | 3,0040 | 7,0693 |
| Treatment Day 14 (SOX9) | Control Negative Day 7 (SOX9) | 5,27500^*^ | ,62406 | ,000 | 3,2424 | 7,3076 |
|  | Control Negative Day 14 (SOX9) | 8,48667^*^ | ,62406 | ,000 | 6,4540 | 10,5193 |
|  | Control Negative Day 21 (SOX9) | 9,00167^*^ | ,62406 | ,000 | 6,9690 | 11,0343 |
|  | Control Positive Day 7 (SOX9) | -,09833 | ,62406 | 1,000 | -2,1310 | 1,9343 |
|  | Control Positive Day 14 (SOX9) | 4,01500^*^ | ,62406 | ,000 | 1,9824 | 6,0476 |
|  | Control Positive Day 21 (SOX9) | 5,22833^*^ | ,62406 | ,000 | 3,1957 | 7,2610 |
|  | Treatment Day 7 (SOX9) | -3,10167^*^ | ,62406 | ,000 | -5,1343 | -1,0690 |
|  | Treatment Day 21 (SOX9 | 1,93500 | ,62406 | ,073 | -,0976 | 3,9676 |
| Treatment Day 21 (SOX9 | Control Negative Day 7 (SOX9) | 3,34000^*^ | ,62406 | ,000 | 1,3074 | 5,3726 |
|  | Control Negative Day 14 (SOX9) | 6,55167^*^ | ,62406 | ,000 | 4,5190 | 8,5843 |
|  | Control Negative Day 21 (SOX9) | 7,06667^*^ | ,62406 | ,000 | 5,0340 | 9,0993 |
|  | Control Positive Day 7 (SOX9) | -2,03333^*^ | ,62406 | ,050 | -4,0660 | -,0007 |
|  | Control Positive Day 14 (SOX9) | 2,08000^*^ | ,62406 | ,041 | ,0474 | 4,1126 |
|  | Control Positive Day 21 (SOX9) | 3,29333^*^ | ,62406 | ,000 | 1,2607 | 5,3260 |
|  | Treatment Day 7 (SOX9) | -5,03667^*^ | ,62406 | ,000 | -7,0693 | -3,0040 |
|  | Treatment Day 14 (SOX9) | -1,93500 | ,62406 | ,073 | -3,9676 | ,0976 |
| *Significant at p<0.05. | | | | | | |
