# Supplementary material for: Osteogenic potential of gingival stromal progenitor cells cultured in platelet rich fibrin is predicted by core-binding factor subunit-α1/Sox9 expression ratio ( in vitro)
Source: F1000Res. 2018 Jul 25;7:1134. [Version 1] doi: 10.12688/f1000research.15423.1 (PMC6097418; doi:10.12688/f1000research.15423.1)
Supplement: Supplementary file 5 [file f1000research-7-16808-s0004.tgz › eb79bb6b-a0d0-46d6-8f5c-167e58ae0aba.docx]

STATISTIC ANALYSIS RAW DATA

| **Descriptives** | | | | | |
| --- | --- | --- | --- | --- | --- |
|  | Group | | | Statistic | Std. Error |
| CBF-alfa1 | Control Negative Day 7 (CBF-alfa1) | Mean | | 8,8867 | ,52065 |
|  |  | 95% Confidence Interval for Mean | Lower Bound | 7,5483 |  |
|  |  |  | Upper Bound | 10,2250 |  |
|  |  | 5% Trimmed Mean | | 8,8741 |  |
|  |  | Median | | 8,8500 |  |
|  |  | Variance | | 1,626 |  |
|  |  | Std. Deviation | | 1,27533 |  |
|  |  | Minimum | | 7,00 |  |
|  |  | Maximum | | 11,00 |  |
|  |  | Range | | 4,00 |  |
|  |  | Interquartile Range | | 1,29 |  |
|  |  | Skewness | | ,391 | ,845 |
|  |  | Kurtosis | | 2,382 | 1,741 |
|  | Control Negative Day 14 (CBF-alfa1) | Mean | | 7,3700 | ,41556 |
|  |  | 95% Confidence Interval for Mean | Lower Bound | 6,3018 |  |
|  |  |  | Upper Bound | 8,4382 |  |
|  |  | 5% Trimmed Mean | | 7,3556 |  |
|  |  | Median | | 7,1200 |  |
|  |  | Variance | | 1,036 |  |
|  |  | Std. Deviation | | 1,01790 |  |
|  |  | Minimum | | 6,00 |  |
|  |  | Maximum | | 9,00 |  |
|  |  | Range | | 3,00 |  |
|  |  | Interquartile Range | | 1,48 |  |
|  |  | Skewness | | ,539 | ,845 |
|  |  | Kurtosis | | ,761 | 1,741 |
|  | Control Negative Day 21 (CBF-alfa1) | Mean | | 3,1867 | ,45939 |
|  |  | 95% Confidence Interval for Mean | Lower Bound | 2,0058 |  |
|  |  |  | Upper Bound | 4,3676 |  |
|  |  | 5% Trimmed Mean | | 3,1519 |  |
|  |  | Median | | 2,9600 |  |
|  |  | Variance | | 1,266 |  |
|  |  | Std. Deviation | | 1,12527 |  |
|  |  | Minimum | | 2,00 |  |
|  |  | Maximum | | 5,00 |  |
|  |  | Range | | 3,00 |  |
|  |  | Interquartile Range | | 2,06 |  |
|  |  | Skewness | | ,815 | ,845 |
|  |  | Kurtosis | | -,131 | 1,741 |
|  | Control Positive Day 7 (CBF-alfa1) | Mean | | 11,0017 | ,26511 |
|  |  | 95% Confidence Interval for Mean | Lower Bound | 10,3202 |  |
|  |  |  | Upper Bound | 11,6832 |  |
|  |  | 5% Trimmed Mean | | 11,0019 |  |
|  |  | Median | | 10,9500 |  |
|  |  | Variance | | ,422 |  |
|  |  | Std. Deviation | | ,64938 |  |
|  |  | Minimum | | 10,00 |  |
|  |  | Maximum | | 12,00 |  |
|  |  | Range | | 2,00 |  |
|  |  | Interquartile Range | | ,82 |  |
|  |  | Skewness | | ,004 | ,845 |
|  |  | Kurtosis | | 1,646 | 1,741 |
|  | Control Positive Day 14 (CBF-alfa1) | Mean | | 9,3883 | ,15779 |
|  |  | 95% Confidence Interval for Mean | Lower Bound | 8,9827 |  |
|  |  |  | Upper Bound | 9,7939 |  |
|  |  | 5% Trimmed Mean | | 9,3759 |  |
|  |  | Median | | 9,3450 |  |
|  |  | Variance | | ,149 |  |
|  |  | Std. Deviation | | ,38649 |  |
|  |  | Minimum | | 9,00 |  |
|  |  | Maximum | | 10,00 |  |
|  |  | Range | | 1,00 |  |
|  |  | Interquartile Range | | ,73 |  |
|  |  | Skewness | | ,660 | ,845 |
|  |  | Kurtosis | | -,304 | 1,741 |
|  | Control Positive Day 21 (CBF-alfa1) | Mean | | 8,3667 | ,31517 |
|  |  | 95% Confidence Interval for Mean | Lower Bound | 7,5565 |  |
|  |  |  | Upper Bound | 9,1768 |  |
|  |  | 5% Trimmed Mean | | 8,4074 |  |
|  |  | Median | | 8,6200 |  |
|  |  | Variance | | ,596 |  |
|  |  | Std. Deviation | | ,77200 |  |
|  |  | Minimum | | 7,00 |  |
|  |  | Maximum | | 9,00 |  |
|  |  | Range | | 2,00 |  |
|  |  | Interquartile Range | | 1,28 |  |
|  |  | Skewness | | -1,348 | ,845 |
|  |  | Kurtosis | | 1,342 | 1,741 |
|  | Treatment Day 7 (CBF-alfa1) | Mean | | 15,1167 | ,93942 |
|  |  | 95% Confidence Interval for Mean | Lower Bound | 12,7018 |  |
|  |  |  | Upper Bound | 17,5315 |  |
|  |  | 5% Trimmed Mean | | 15,0374 |  |
|  |  | Median | | 15,0000 |  |
|  |  | Variance | | 5,295 |  |
|  |  | Std. Deviation | | 2,30111 |  |
|  |  | Minimum | | 12,66 |  |
|  |  | Maximum | | 19,00 |  |
|  |  | Range | | 6,34 |  |
|  |  | Interquartile Range | | 3,87 |  |
|  |  | Skewness | | ,863 | ,845 |
|  |  | Kurtosis | | ,856 | 1,741 |
|  | Treatment Day 14 (CBF-alfa1) | Mean | | 12,0100 | ,57837 |
|  |  | 95% Confidence Interval for Mean | Lower Bound | 10,5233 |  |
|  |  |  | Upper Bound | 13,4967 |  |
|  |  | 5% Trimmed Mean | | 12,0111 |  |
|  |  | Median | | 12,0650 |  |
|  |  | Variance | | 2,007 |  |
|  |  | Std. Deviation | | 1,41670 |  |
|  |  | Minimum | | 10,00 |  |
|  |  | Maximum | | 14,00 |  |
|  |  | Range | | 4,00 |  |
|  |  | Interquartile Range | | 2,45 |  |
|  |  | Skewness | | -,049 | ,845 |
|  |  | Kurtosis | | -,380 | 1,741 |
|  | Treatment Day 21 (CBF-alfa1) | Mean | | 10,5517 | ,40497 |
|  |  | 95% Confidence Interval for Mean | Lower Bound | 9,5107 |  |
|  |  |  | Upper Bound | 11,5927 |  |
|  |  | 5% Trimmed Mean | | 10,5574 |  |
|  |  | Median | | 10,5850 |  |
|  |  | Variance | | ,984 |  |
|  |  | Std. Deviation | | ,99198 |  |
|  |  | Minimum | | 9,00 |  |
|  |  | Maximum | | 12,00 |  |
|  |  | Range | | 3,00 |  |
|  |  | Interquartile Range | | 1,39 |  |
|  |  | Skewness | | -,207 | ,845 |
|  |  | Kurtosis | | 1,173 | 1,741 |

Normality Test

| **Tests of Normality** | | | | | | | |
| --- | --- | --- | --- | --- | --- | --- | --- |
|  | Group | Kolmogorov-Smirnov^a^ | | | Shapiro-Wilk | | |
|  |  | Statistic | df | Sig. | Statistic | df | Sig. |
| CBF-alfa1 | Control Negative Day 7 (CBF-alfa1) | ,298 | 6 | ,104 | ,894 | 6 | ,339 |
|  | Control Negative Day 14 (CBF-alfa1) | ,233 | 6 | ,200^*^ | ,950 | 6 | ,738 |
|  | Control Negative Day 21 (CBF-alfa1) | ,233 | 6 | ,200^*^ | ,928 | 6 | ,568 |
|  | Control Positive Day 7 (CBF-alfa1) | ,235 | 6 | ,200^*^ | ,950 | 6 | ,742 |
|  | Control Positive Day 14 (CBF-alfa1) | ,176 | 6 | ,200^*^ | ,924 | 6 | ,532 |
|  | Control Positive Day 21 (CBF-alfa1) | ,250 | 6 | ,200^*^ | ,852 | 6 | ,165 |
|  | Treatment Day 7 (CBF-alfa1) | ,187 | 6 | ,200^*^ | ,918 | 6 | ,491 |
|  | Treatment Day 14 (CBF-alfa1) | ,102 | 6 | ,200^*^ | ,998 | 6 | 1,000 |
|  | Treatment Day 21 (CBF-alfa1) | ,172 | 6 | ,200^*^ | ,980 | 6 | ,952 |
| *. This is a lower bound of the true significance. | | | | | | | |
| a. Lilliefors Significance Correction | | | | | | | |

Homogenity Test

| **Test of Homogeneity of Variances** | | | | | |
| --- | --- | --- | --- | --- | --- |
|  | | Levene Statistic | df1 | df2 | Sig. |
| CBF-alfa1 | Based on Mean | 1,510 | 8 | 45 | ,181 |
|  | Based on Median | 1,291 | 8 | 45 | ,273 |
|  | Based on Median and with adjusted df | 1,291 | 8 | 23,102 | ,296 |
|  | Based on trimmed mean | 1,417 | 8 | 45 | ,216 |

ANOVA Test Result

| **ANOVA** | | | | | |
| --- | --- | --- | --- | --- | --- |
| CBF-alfa1 | | | | | |
|  | Sum of Squares | df | Mean Square | F | Sig. |
| Between Groups | 523,562 | 8 | 65,445 | 44,015 | ,000 |
| Within Groups | 66,910 | 45 | 1,487 |  |  |
| Total | 590,472 | 53 |  |  |  |

**MULTIPLE COMPARISON**

| **Multiple Comparisons** | | | | | | |
| --- | --- | --- | --- | --- | --- | --- |
| Dependent Variable: CBF-alfa1 | | | | | | |
| Tukey HSD | | | | | | |
| (I) Group | (J) Group | Mean Difference (I-J) | Std. Error | Sig. | 95% Confidence Interval | |
|  |  |  |  |  | Lower Bound | Upper Bound |
| Control Negative Day 7 (CBF-alfa1) | Control Negative Day 14 (CBF-alfa1) | 1,51667 | ,70401 | ,452 | -,7764 | 3,8097 |
|  | Control Negative Day 21 (CBF-alfa1) | 5,70000^*^ | ,70401 | ,000 | 3,4069 | 7,9931 |
|  | Control Positive Day 7 (CBF-alfa1) | -2,11500 | ,70401 | ,092 | -4,4081 | ,1781 |
|  | Control Positive Day 14 (CBF-alfa1) | -,50167 | ,70401 | ,998 | -2,7947 | 1,7914 |
|  | Control Positive Day 21 (CBF-alfa1) | ,52000 | ,70401 | ,998 | -1,7731 | 2,8131 |
|  | Treatment Day 7 (CBF-alfa1) | -6,23000^*^ | ,70401 | ,000 | -8,5231 | -3,9369 |
|  | Treatment Day 14 (CBF-alfa1) | -3,12333^*^ | ,70401 | ,002 | -5,4164 | -,8303 |
|  | Treatment Day 21 (CBF-alfa1) | -1,66500 | ,70401 | ,327 | -3,9581 | ,6281 |
| Control Negative Day 14 (CBF-alfa1) | Control Negative Day 7 (CBF-alfa1) | -1,51667 | ,70401 | ,452 | -3,8097 | ,7764 |
|  | Control Negative Day 21 (CBF-alfa1) | 4,18333^*^ | ,70401 | ,000 | 1,8903 | 6,4764 |
|  | Control Positive Day 7 (CBF-alfa1) | -3,63167^*^ | ,70401 | ,000 | -5,9247 | -1,3386 |
|  | Control Positive Day 14 (CBF-alfa1) | -2,01833 | ,70401 | ,125 | -4,3114 | ,2747 |
|  | Control Positive Day 21 (CBF-alfa1) | -,99667 | ,70401 | ,886 | -3,2897 | 1,2964 |
|  | Treatment Day 7 (CBF-alfa1) | -7,74667^*^ | ,70401 | ,000 | -10,0397 | -5,4536 |
|  | Treatment Day 14 (CBF-alfa1) | -4,64000^*^ | ,70401 | ,000 | -6,9331 | -2,3469 |
|  | Treatment Day 21 (CBF-alfa1) | -3,18167^*^ | ,70401 | ,001 | -5,4747 | -,8886 |
| Control Negative Day 21 (CBF-alfa1) | Control Negative Day 7 (CBF-alfa1) | -5,70000^*^ | ,70401 | ,000 | -7,9931 | -3,4069 |
|  | Control Negative Day 14 (CBF-alfa1) | -4,18333^*^ | ,70401 | ,000 | -6,4764 | -1,8903 |
|  | Control Positive Day 7 (CBF-alfa1) | -7,81500^*^ | ,70401 | ,000 | -10,1081 | -5,5219 |
|  | Control Positive Day 14 (CBF-alfa1) | -6,20167^*^ | ,70401 | ,000 | -8,4947 | -3,9086 |
|  | Control Positive Day 21 (CBF-alfa1) | -5,18000^*^ | ,70401 | ,000 | -7,4731 | -2,8869 |
|  | Treatment Day 7 (CBF-alfa1) | -11,93000^*^ | ,70401 | ,000 | -14,2231 | -9,6369 |
|  | Treatment Day 14 (CBF-alfa1) | -8,82333^*^ | ,70401 | ,000 | -11,1164 | -6,5303 |
|  | Treatment Day 21 (CBF-alfa1) | -7,36500^*^ | ,70401 | ,000 | -9,6581 | -5,0719 |
| Control Positive Day 7 (CBF-alfa1) | Control Negative Day 7 (CBF-alfa1) | 2,11500 | ,70401 | ,092 | -,1781 | 4,4081 |
|  | Control Negative Day 14 (CBF-alfa1) | 3,63167^*^ | ,70401 | ,000 | 1,3386 | 5,9247 |
|  | Control Negative Day 21 (CBF-alfa1) | 7,81500^*^ | ,70401 | ,000 | 5,5219 | 10,1081 |
|  | Control Positive Day 14 (CBF-alfa1) | 1,61333 | ,70401 | ,369 | -,6797 | 3,9064 |
|  | Control Positive Day 21 (CBF-alfa1) | 2,63500^*^ | ,70401 | ,014 | ,3419 | 4,9281 |
|  | Treatment Day 7 (CBF-alfa1) | -4,11500^*^ | ,70401 | ,000 | -6,4081 | -1,8219 |
|  | Treatment Day 14 (CBF-alfa1) | -1,00833 | ,70401 | ,879 | -3,3014 | 1,2847 |
|  | Treatment Day 21 (CBF-alfa1) | ,45000 | ,70401 | ,999 | -1,8431 | 2,7431 |
| Control Positive Day 14 (CBF-alfa1) | Control Negative Day 7 (CBF-alfa1) | ,50167 | ,70401 | ,998 | -1,7914 | 2,7947 |
|  | Control Negative Day 14 (CBF-alfa1) | 2,01833 | ,70401 | ,125 | -,2747 | 4,3114 |
|  | Control Negative Day 21 (CBF-alfa1) | 6,20167^*^ | ,70401 | ,000 | 3,9086 | 8,4947 |
|  | Control Positive Day 7 (CBF-alfa1) | -1,61333 | ,70401 | ,369 | -3,9064 | ,6797 |
|  | Control Positive Day 21 (CBF-alfa1) | 1,02167 | ,70401 | ,871 | -1,2714 | 3,3147 |
|  | Treatment Day 7 (CBF-alfa1) | -5,72833^*^ | ,70401 | ,000 | -8,0214 | -3,4353 |
|  | Treatment Day 14 (CBF-alfa1) | -2,62167^*^ | ,70401 | ,015 | -4,9147 | -,3286 |
|  | Treatment Day 21 (CBF-alfa1) | -1,16333 | ,70401 | ,771 | -3,4564 | 1,1297 |
| Control Positive Day 21 (CBF-alfa1) | Control Negative Day 7 (CBF-alfa1) | -,52000 | ,70401 | ,998 | -2,8131 | 1,7731 |
|  | Control Negative Day 14 (CBF-alfa1) | ,99667 | ,70401 | ,886 | -1,2964 | 3,2897 |
|  | Control Negative Day 21 (CBF-alfa1) | 5,18000^*^ | ,70401 | ,000 | 2,8869 | 7,4731 |
|  | Control Positive Day 7 (CBF-alfa1) | -2,63500^*^ | ,70401 | ,014 | -4,9281 | -,3419 |
|  | Control Positive Day 14 (CBF-alfa1) | -1,02167 | ,70401 | ,871 | -3,3147 | 1,2714 |
|  | Treatment Day 7 (CBF-alfa1) | -6,75000^*^ | ,70401 | ,000 | -9,0431 | -4,4569 |
|  | Treatment Day 14 (CBF-alfa1) | -3,64333^*^ | ,70401 | ,000 | -5,9364 | -1,3503 |
|  | Treatment Day 21 (CBF-alfa1) | -2,18500 | ,70401 | ,073 | -4,4781 | ,1081 |
| Treatment Day 7 (CBF-alfa1) | Control Negative Day 7 (CBF-alfa1) | 6,23000^*^ | ,70401 | ,000 | 3,9369 | 8,5231 |
|  | Control Negative Day 14 (CBF-alfa1) | 7,74667^*^ | ,70401 | ,000 | 5,4536 | 10,0397 |
|  | Control Negative Day 21 (CBF-alfa1) | 11,93000^*^ | ,70401 | ,000 | 9,6369 | 14,2231 |
|  | Control Positive Day 7 (CBF-alfa1) | 4,11500^*^ | ,70401 | ,000 | 1,8219 | 6,4081 |
|  | Control Positive Day 14 (CBF-alfa1) | 5,72833^*^ | ,70401 | ,000 | 3,4353 | 8,0214 |
|  | Control Positive Day 21 (CBF-alfa1) | 6,75000^*^ | ,70401 | ,000 | 4,4569 | 9,0431 |
|  | Treatment Day 14 (CBF-alfa1) | 3,10667^*^ | ,70401 | ,002 | ,8136 | 5,3997 |
|  | Treatment Day 21 (CBF-alfa1) | 4,56500^*^ | ,70401 | ,000 | 2,2719 | 6,8581 |
| Treatment Day 14 (CBF-alfa1) | Control Negative Day 7 (CBF-alfa1) | 3,12333^*^ | ,70401 | ,002 | ,8303 | 5,4164 |
|  | Control Negative Day 14 (CBF-alfa1) | 4,64000^*^ | ,70401 | ,000 | 2,3469 | 6,9331 |
|  | Control Negative Day 21 (CBF-alfa1) | 8,82333^*^ | ,70401 | ,000 | 6,5303 | 11,1164 |
|  | Control Positive Day 7 (CBF-alfa1) | 1,00833 | ,70401 | ,879 | -1,2847 | 3,3014 |
|  | Control Positive Day 14 (CBF-alfa1) | 2,62167^*^ | ,70401 | ,015 | ,3286 | 4,9147 |
|  | Control Positive Day 21 (CBF-alfa1) | 3,64333^*^ | ,70401 | ,000 | 1,3503 | 5,9364 |
|  | Treatment Day 7 (CBF-alfa1) | -3,10667^*^ | ,70401 | ,002 | -5,3997 | -,8136 |
|  | Treatment Day 21 (CBF-alfa1) | 1,45833 | ,70401 | ,505 | -,8347 | 3,7514 |
| Treatment Day 21 (CBF-alfa1) | Control Negative Day 7 (CBF-alfa1) | 1,66500 | ,70401 | ,327 | -,6281 | 3,9581 |
|  | Control Negative Day 14 (CBF-alfa1) | 3,18167^*^ | ,70401 | ,001 | ,8886 | 5,4747 |
|  | Control Negative Day 21 (CBF-alfa1) | 7,36500^*^ | ,70401 | ,000 | 5,0719 | 9,6581 |
|  | Control Positive Day 7 (CBF-alfa1) | -,45000 | ,70401 | ,999 | -2,7431 | 1,8431 |
|  | Control Positive Day 14 (CBF-alfa1) | 1,16333 | ,70401 | ,771 | -1,1297 | 3,4564 |
|  | Control Positive Day 21 (CBF-alfa1) | 2,18500 | ,70401 | ,073 | -,1081 | 4,4781 |
|  | Treatment Day 7 (CBF-alfa1) | -4,56500^*^ | ,70401 | ,000 | -6,8581 | -2,2719 |
|  | Treatment Day 14 (CBF-alfa1) | -1,45833 | ,70401 | ,505 | -3,7514 | ,8347 |
| *. The mean difference is significant at the 0.05 level. | | | | | | |

| **CBF-alfa1** | | | | | | | |
| --- | --- | --- | --- | --- | --- | --- | --- |
| Tukey HSD^a^ | | | | | | | |
| Group | N | Subset for alpha = 0.05 | | | | | |
|  |  | 1 | 2 | 3 | 4 | 5 | 6 |
| Control Negative Day 21 (CBF-alfa1) | 6 | 3,1867 |  |  |  |  |  |
| Control Negative Day 14 (CBF-alfa1) | 6 |  | 7,3700 |  |  |  |  |
| Control Positive Day 21 (CBF-alfa1) | 6 |  | 8,3667 | 8,3667 |  |  |  |
| Control Negative Day 7 (CBF-alfa1) | 6 |  | 8,8867 | 8,8867 | 8,8867 |  |  |
| Control Positive Day 14 (CBF-alfa1) | 6 |  | 9,3883 | 9,3883 | 9,3883 |  |  |
| Treatment Day 21 (CBF-alfa1) | 6 |  |  | 10,5517 | 10,5517 | 10,5517 |  |
| Control Positive Day 7 (CBF-alfa1) | 6 |  |  |  | 11,0017 | 11,0017 |  |
| Treatment Day 14 (CBF-alfa1) | 6 |  |  |  |  | 12,0100 |  |
| Treatment Day 7 (CBF-alfa1) | 6 |  |  |  |  |  | 15,1167 |
| Sig. |  | 1,000 | ,125 | ,073 | ,092 | ,505 | 1,000 |
| Means for groups in homogeneous subsets are displayed. | | | | | | | |
| a. Uses Harmonic Mean Sample Size = 6,000. | | | | | | | |
