# Supplementary material for: Osteogenic potential of gingival stromal progenitor cells cultured in platelet rich fibrin is predicted by core-binding factor subunit-α1/Sox9 expression ratio ( in vitro)
Source: F1000Res. 2018 Jul 25;7:1134. [Version 1] doi: 10.12688/f1000research.15423.1 (PMC6097418; doi:10.12688/f1000research.15423.1)
Supplement: Supplementary file 6 [file f1000research-7-16808-s0005.tgz › 8d679ea1-eb7a-48fb-84a8-d2e536af23e5.docx]

SOX9 Analysis

| **Descriptives** | | | | | |
| --- | --- | --- | --- | --- | --- |
|  | Group | | | Statistic | Std. Error |
| SOX9 | Control Negative Day 7 (SOX9) | Mean | | 5,8433 | ,34612 |
|  |  | 95% Confidence Interval for Mean | Lower Bound | 4,9536 |  |
|  |  |  | Upper Bound | 6,7331 |  |
|  |  | 5% Trimmed Mean | | 5,8259 |  |
|  |  | Median | | 5,8200 |  |
|  |  | Variance | | ,719 |  |
|  |  | Std. Deviation | | ,84781 |  |
|  |  | Minimum | | 5,00 |  |
|  |  | Maximum | | 7,00 |  |
|  |  | Range | | 2,00 |  |
|  |  | Interquartile Range | | 1,56 |  |
|  |  | Skewness | | ,248 | ,845 |
|  |  | Kurtosis | | -2,133 | 1,741 |
|  | Control Negative Day 14 (SOX9) | Mean | | 2,6317 | ,16778 |
|  |  | 95% Confidence Interval for Mean | Lower Bound | 2,2004 |  |
|  |  |  | Upper Bound | 3,0630 |  |
|  |  | 5% Trimmed Mean | | 2,6463 |  |
|  |  | Median | | 2,7650 |  |
|  |  | Variance | | ,169 |  |
|  |  | Std. Deviation | | ,41097 |  |
|  |  | Minimum | | 2,00 |  |
|  |  | Maximum | | 3,00 |  |
|  |  | Range | | 1,00 |  |
|  |  | Interquartile Range | | ,81 |  |
|  |  | Skewness | | -,858 | ,845 |
|  |  | Kurtosis | | -,955 | 1,741 |
|  | Control Negative Day 21 (SOX9) | Mean | | 2,1167 | ,33034 |
|  |  | 95% Confidence Interval for Mean | Lower Bound | 1,2675 |  |
|  |  |  | Upper Bound | 2,9658 |  |
|  |  | 5% Trimmed Mean | | 2,1296 |  |
|  |  | Median | | 2,0850 |  |
|  |  | Variance | | ,655 |  |
|  |  | Std. Deviation | | ,80916 |  |
|  |  | Minimum | | 1,00 |  |
|  |  | Maximum | | 3,00 |  |
|  |  | Range | | 2,00 |  |
|  |  | Interquartile Range | | 1,60 |  |
|  |  | Skewness | | -,129 | ,845 |
|  |  | Kurtosis | | -1,516 | 1,741 |
|  | Control Positive Day 7 (SOX9) | Mean | | 11,2167 | ,42273 |
|  |  | 95% Confidence Interval for Mean | Lower Bound | 10,1300 |  |
|  |  |  | Upper Bound | 12,3033 |  |
|  |  | 5% Trimmed Mean | | 11,1852 |  |
|  |  | Median | | 11,1200 |  |
|  |  | Variance | | 1,072 |  |
|  |  | Std. Deviation | | 1,03546 |  |
|  |  | Minimum | | 10,00 |  |
|  |  | Maximum | | 13,00 |  |
|  |  | Range | | 3,00 |  |
|  |  | Interquartile Range | | 1,56 |  |
|  |  | Skewness | | ,951 | ,845 |
|  |  | Kurtosis | | 1,424 | 1,741 |
|  | Control Positive Day 14 (SOX9) | Mean | | 7,1033 | ,44993 |
|  |  | 95% Confidence Interval for Mean | Lower Bound | 5,9467 |  |
|  |  |  | Upper Bound | 8,2599 |  |
|  |  | 5% Trimmed Mean | | 7,0593 |  |
|  |  | Median | | 7,0300 |  |
|  |  | Variance | | 1,215 |  |
|  |  | Std. Deviation | | 1,10210 |  |
|  |  | Minimum | | 6,00 |  |
|  |  | Maximum | | 9,00 |  |
|  |  | Range | | 3,00 |  |
|  |  | Interquartile Range | | 1,83 |  |
|  |  | Skewness | | ,987 | ,845 |
|  |  | Kurtosis | | 1,167 | 1,741 |
|  | Control Positive Day 21 (SOX9) | Mean | | 5,8900 | ,29018 |
|  |  | 95% Confidence Interval for Mean | Lower Bound | 5,1441 |  |
|  |  |  | Upper Bound | 6,6359 |  |
|  |  | 5% Trimmed Mean | | 5,8778 |  |
|  |  | Median | | 5,8000 |  |
|  |  | Variance | | ,505 |  |
|  |  | Std. Deviation | | ,71080 |  |
|  |  | Minimum | | 5,00 |  |
|  |  | Maximum | | 7,00 |  |
|  |  | Range | | 2,00 |  |
|  |  | Interquartile Range | | 1,18 |  |
|  |  | Skewness | | ,514 | ,845 |
|  |  | Kurtosis | | -,119 | 1,741 |
|  | Treatment Day 7 (SOX9) | Mean | | 14,2200 | ,72970 |
|  |  | 95% Confidence Interval for Mean | Lower Bound | 12,3443 |  |
|  |  |  | Upper Bound | 16,0957 |  |
|  |  | 5% Trimmed Mean | | 14,1889 |  |
|  |  | Median | | 14,0800 |  |
|  |  | Variance | | 3,195 |  |
|  |  | Std. Deviation | | 1,78739 |  |
|  |  | Minimum | | 12,00 |  |
|  |  | Maximum | | 17,00 |  |
|  |  | Range | | 5,00 |  |
|  |  | Interquartile Range | | 3,15 |  |
|  |  | Skewness | | ,482 | ,845 |
|  |  | Kurtosis | | -,105 | 1,741 |
|  | Treatment Day 14 (SOX9) | Mean | | 11,1183 | ,46877 |
|  |  | 95% Confidence Interval for Mean | Lower Bound | 9,9133 |  |
|  |  |  | Upper Bound | 12,3233 |  |
|  |  | 5% Trimmed Mean | | 11,0759 |  |
|  |  | Median | | 10,7150 |  |
|  |  | Variance | | 1,318 |  |
|  |  | Std. Deviation | | 1,14824 |  |
|  |  | Minimum | | 10,00 |  |
|  |  | Maximum | | 13,00 |  |
|  |  | Range | | 3,00 |  |
|  |  | Interquartile Range | | 1,96 |  |
|  |  | Skewness | | ,995 | ,845 |
|  |  | Kurtosis | | -,133 | 1,741 |
|  | Treatment Day 21 (SOX9 | Mean | | 9,1833 | ,52717 |
|  |  | 95% Confidence Interval for Mean | Lower Bound | 7,8282 |  |
|  |  |  | Upper Bound | 10,5385 |  |
|  |  | 5% Trimmed Mean | | 9,2037 |  |
|  |  | Median | | 9,2400 |  |
|  |  | Variance | | 1,667 |  |
|  |  | Std. Deviation | | 1,29129 |  |
|  |  | Minimum | | 7,00 |  |
|  |  | Maximum | | 11,00 |  |
|  |  | Range | | 4,00 |  |
|  |  | Interquartile Range | | 1,46 |  |
|  |  | Skewness | | -,603 | ,845 |
|  |  | Kurtosis | | 2,223 | 1,741 |

Normality Test

| **Tests of Normality** | | | | | | | |
| --- | --- | --- | --- | --- | --- | --- | --- |
|  | Group | Kolmogorov-Smirnov^a^ | | | Shapiro-Wilk | | |
|  |  | Statistic | df | Sig. | Statistic | df | Sig. |
| SOX9 | Control Negative Day 7 (SOX9) | ,231 | 6 | ,200^*^ | ,874 | 6 | ,245 |
|  | Control Negative Day 14 (SOX9) | ,280 | 6 | ,154 | ,862 | 6 | ,197 |
|  | Control Negative Day 21 (SOX9) | ,196 | 6 | ,200^*^ | ,927 | 6 | ,555 |
|  | Control Positive Day 7 (SOX9) | ,200 | 6 | ,200^*^ | ,947 | 6 | ,717 |
|  | Control Positive Day 14 (SOX9) | ,193 | 6 | ,200^*^ | ,900 | 6 | ,376 |
|  | Control Positive Day 21 (SOX9) | ,158 | 6 | ,200^*^ | ,981 | 6 | ,954 |
|  | Treatment Day 7 (SOX9) | ,180 | 6 | ,200^*^ | ,973 | 6 | ,911 |
|  | Treatment Day 14 (SOX9) | ,226 | 6 | ,200^*^ | ,899 | 6 | ,366 |
|  | Treatment Day 21 (SOX9 | ,277 | 6 | ,167 | ,921 | 6 | ,511 |
| *. This is a lower bound of the true significance. | | | | | | | |
| a. Lilliefors Significance Correction | | | | | | | |

Homogenity Test

| **Test of Homogeneity of Variances** | | | | | |
| --- | --- | --- | --- | --- | --- |
|  | | Levene Statistic | df1 | df2 | Sig. |
| SOX9 | Based on Mean | 1,015 | 8 | 45 | ,439 |
|  | Based on Median | ,902 | 8 | 45 | ,523 |
|  | Based on Median and with adjusted df | ,902 | 8 | 26,869 | ,529 |
|  | Based on trimmed mean | ,967 | 8 | 45 | ,473 |

ANOVA Test Result

| **ANOVA** | | | | | |
| --- | --- | --- | --- | --- | --- |
| SOX9 | | | | | |
|  | Sum of Squares | df | Mean Square | F | Sig. |
| Between Groups | 796,221 | 8 | 99,528 | 85,187 | ,000 |
| Within Groups | 52,576 | 45 | 1,168 |  |  |
| Total | 848,797 | 53 |  |  |  |

**MULTIPLE COMPARISON**

| **Multiple Comparisons** | | | | | | |
| --- | --- | --- | --- | --- | --- | --- |
| Dependent Variable: SOX9 | | | | | | |
| Tukey HSD | | | | | | |
| (I) Group | (J) Group | Mean Difference (I-J) | Std. Error | Sig. | 95% Confidence Interval | |
|  |  |  |  |  | Lower Bound | Upper Bound |
| Control Negative Day 7 (SOX9) | Control Negative Day 14 (SOX9) | 3,21167^*^ | ,62406 | ,000 | 1,1790 | 5,2443 |
|  | Control Negative Day 21 (SOX9) | 3,72667^*^ | ,62406 | ,000 | 1,6940 | 5,7593 |
|  | Control Positive Day 7 (SOX9) | -5,37333^*^ | ,62406 | ,000 | -7,4060 | -3,3407 |
|  | Control Positive Day 14 (SOX9) | -1,26000 | ,62406 | ,539 | -3,2926 | ,7726 |
|  | Control Positive Day 21 (SOX9) | -,04667 | ,62406 | 1,000 | -2,0793 | 1,9860 |
|  | Treatment Day 7 (SOX9) | -8,37667^*^ | ,62406 | ,000 | -10,4093 | -6,3440 |
|  | Treatment Day 14 (SOX9) | -5,27500^*^ | ,62406 | ,000 | -7,3076 | -3,2424 |
|  | Treatment Day 21 (SOX9 | -3,34000^*^ | ,62406 | ,000 | -5,3726 | -1,3074 |
| Control Negative Day 14 (SOX9) | Control Negative Day 7 (SOX9) | -3,21167^*^ | ,62406 | ,000 | -5,2443 | -1,1790 |
|  | Control Negative Day 21 (SOX9) | ,51500 | ,62406 | ,995 | -1,5176 | 2,5476 |
|  | Control Positive Day 7 (SOX9) | -8,58500^*^ | ,62406 | ,000 | -10,6176 | -6,5524 |
|  | Control Positive Day 14 (SOX9) | -4,47167^*^ | ,62406 | ,000 | -6,5043 | -2,4390 |
|  | Control Positive Day 21 (SOX9) | -3,25833^*^ | ,62406 | ,000 | -5,2910 | -1,2257 |
|  | Treatment Day 7 (SOX9) | -11,58833^*^ | ,62406 | ,000 | -13,6210 | -9,5557 |
|  | Treatment Day 14 (SOX9) | -8,48667^*^ | ,62406 | ,000 | -10,5193 | -6,4540 |
|  | Treatment Day 21 (SOX9 | -6,55167^*^ | ,62406 | ,000 | -8,5843 | -4,5190 |
| Control Negative Day 21 (SOX9) | Control Negative Day 7 (SOX9) | -3,72667^*^ | ,62406 | ,000 | -5,7593 | -1,6940 |
|  | Control Negative Day 14 (SOX9) | -,51500 | ,62406 | ,995 | -2,5476 | 1,5176 |
|  | Control Positive Day 7 (SOX9) | -9,10000^*^ | ,62406 | ,000 | -11,1326 | -7,0674 |
|  | Control Positive Day 14 (SOX9) | -4,98667^*^ | ,62406 | ,000 | -7,0193 | -2,9540 |
|  | Control Positive Day 21 (SOX9) | -3,77333^*^ | ,62406 | ,000 | -5,8060 | -1,7407 |
|  | Treatment Day 7 (SOX9) | -12,10333^*^ | ,62406 | ,000 | -14,1360 | -10,0707 |
|  | Treatment Day 14 (SOX9) | -9,00167^*^ | ,62406 | ,000 | -11,0343 | -6,9690 |
|  | Treatment Day 21 (SOX9 | -7,06667^*^ | ,62406 | ,000 | -9,0993 | -5,0340 |
| Control Positive Day 7 (SOX9) | Control Negative Day 7 (SOX9) | 5,37333^*^ | ,62406 | ,000 | 3,3407 | 7,4060 |
|  | Control Negative Day 14 (SOX9) | 8,58500^*^ | ,62406 | ,000 | 6,5524 | 10,6176 |
|  | Control Negative Day 21 (SOX9) | 9,10000^*^ | ,62406 | ,000 | 7,0674 | 11,1326 |
|  | Control Positive Day 14 (SOX9) | 4,11333^*^ | ,62406 | ,000 | 2,0807 | 6,1460 |
|  | Control Positive Day 21 (SOX9) | 5,32667^*^ | ,62406 | ,000 | 3,2940 | 7,3593 |
|  | Treatment Day 7 (SOX9) | -3,00333^*^ | ,62406 | ,001 | -5,0360 | -,9707 |
|  | Treatment Day 14 (SOX9) | ,09833 | ,62406 | 1,000 | -1,9343 | 2,1310 |
|  | Treatment Day 21 (SOX9 | 2,03333^*^ | ,62406 | ,050 | ,0007 | 4,0660 |
| Control Positive Day 14 (SOX9) | Control Negative Day 7 (SOX9) | 1,26000 | ,62406 | ,539 | -,7726 | 3,2926 |
|  | Control Negative Day 14 (SOX9) | 4,47167^*^ | ,62406 | ,000 | 2,4390 | 6,5043 |
|  | Control Negative Day 21 (SOX9) | 4,98667^*^ | ,62406 | ,000 | 2,9540 | 7,0193 |
|  | Control Positive Day 7 (SOX9) | -4,11333^*^ | ,62406 | ,000 | -6,1460 | -2,0807 |
|  | Control Positive Day 21 (SOX9) | 1,21333 | ,62406 | ,588 | -,8193 | 3,2460 |
|  | Treatment Day 7 (SOX9) | -7,11667^*^ | ,62406 | ,000 | -9,1493 | -5,0840 |
|  | Treatment Day 14 (SOX9) | -4,01500^*^ | ,62406 | ,000 | -6,0476 | -1,9824 |
|  | Treatment Day 21 (SOX9 | -2,08000^*^ | ,62406 | ,041 | -4,1126 | -,0474 |
| Control Positive Day 21 (SOX9) | Control Negative Day 7 (SOX9) | ,04667 | ,62406 | 1,000 | -1,9860 | 2,0793 |
|  | Control Negative Day 14 (SOX9) | 3,25833^*^ | ,62406 | ,000 | 1,2257 | 5,2910 |
|  | Control Negative Day 21 (SOX9) | 3,77333^*^ | ,62406 | ,000 | 1,7407 | 5,8060 |
|  | Control Positive Day 7 (SOX9) | -5,32667^*^ | ,62406 | ,000 | -7,3593 | -3,2940 |
|  | Control Positive Day 14 (SOX9) | -1,21333 | ,62406 | ,588 | -3,2460 | ,8193 |
|  | Treatment Day 7 (SOX9) | -8,33000^*^ | ,62406 | ,000 | -10,3626 | -6,2974 |
|  | Treatment Day 14 (SOX9) | -5,22833^*^ | ,62406 | ,000 | -7,2610 | -3,1957 |
|  | Treatment Day 21 (SOX9 | -3,29333^*^ | ,62406 | ,000 | -5,3260 | -1,2607 |
| Treatment Day 7 (SOX9) | Control Negative Day 7 (SOX9) | 8,37667^*^ | ,62406 | ,000 | 6,3440 | 10,4093 |
|  | Control Negative Day 14 (SOX9) | 11,58833^*^ | ,62406 | ,000 | 9,5557 | 13,6210 |
|  | Control Negative Day 21 (SOX9) | 12,10333^*^ | ,62406 | ,000 | 10,0707 | 14,1360 |
|  | Control Positive Day 7 (SOX9) | 3,00333^*^ | ,62406 | ,001 | ,9707 | 5,0360 |
|  | Control Positive Day 14 (SOX9) | 7,11667^*^ | ,62406 | ,000 | 5,0840 | 9,1493 |
|  | Control Positive Day 21 (SOX9) | 8,33000^*^ | ,62406 | ,000 | 6,2974 | 10,3626 |
|  | Treatment Day 14 (SOX9) | 3,10167^*^ | ,62406 | ,000 | 1,0690 | 5,1343 |
|  | Treatment Day 21 (SOX9 | 5,03667^*^ | ,62406 | ,000 | 3,0040 | 7,0693 |
| Treatment Day 14 (SOX9) | Control Negative Day 7 (SOX9) | 5,27500^*^ | ,62406 | ,000 | 3,2424 | 7,3076 |
|  | Control Negative Day 14 (SOX9) | 8,48667^*^ | ,62406 | ,000 | 6,4540 | 10,5193 |
|  | Control Negative Day 21 (SOX9) | 9,00167^*^ | ,62406 | ,000 | 6,9690 | 11,0343 |
|  | Control Positive Day 7 (SOX9) | -,09833 | ,62406 | 1,000 | -2,1310 | 1,9343 |
|  | Control Positive Day 14 (SOX9) | 4,01500^*^ | ,62406 | ,000 | 1,9824 | 6,0476 |
|  | Control Positive Day 21 (SOX9) | 5,22833^*^ | ,62406 | ,000 | 3,1957 | 7,2610 |
|  | Treatment Day 7 (SOX9) | -3,10167^*^ | ,62406 | ,000 | -5,1343 | -1,0690 |
|  | Treatment Day 21 (SOX9 | 1,93500 | ,62406 | ,073 | -,0976 | 3,9676 |
| Treatment Day 21 (SOX9 | Control Negative Day 7 (SOX9) | 3,34000^*^ | ,62406 | ,000 | 1,3074 | 5,3726 |
|  | Control Negative Day 14 (SOX9) | 6,55167^*^ | ,62406 | ,000 | 4,5190 | 8,5843 |
|  | Control Negative Day 21 (SOX9) | 7,06667^*^ | ,62406 | ,000 | 5,0340 | 9,0993 |
|  | Control Positive Day 7 (SOX9) | -2,03333^*^ | ,62406 | ,050 | -4,0660 | -,0007 |
|  | Control Positive Day 14 (SOX9) | 2,08000^*^ | ,62406 | ,041 | ,0474 | 4,1126 |
|  | Control Positive Day 21 (SOX9) | 3,29333^*^ | ,62406 | ,000 | 1,2607 | 5,3260 |
|  | Treatment Day 7 (SOX9) | -5,03667^*^ | ,62406 | ,000 | -7,0693 | -3,0040 |
|  | Treatment Day 14 (SOX9) | -1,93500 | ,62406 | ,073 | -3,9676 | ,0976 |
| *. The mean difference is significant at the 0.05 level. | | | | | | |

| **SOX9** | | | | | | |
| --- | --- | --- | --- | --- | --- | --- |
| Tukey HSD^a^ | | | | | | |
| Group | N | Subset for alpha = 0.05 | | | | |
|  |  | 1 | 2 | 3 | 4 | 5 |
| Control Negative Day 21 (SOX9) | 6 | 2,1167 |  |  |  |  |
| Control Negative Day 14 (SOX9) | 6 | 2,6317 |  |  |  |  |
| Control Negative Day 7 (SOX9) | 6 |  | 5,8433 |  |  |  |
| Control Positive Day 21 (SOX9) | 6 |  | 5,8900 |  |  |  |
| Control Positive Day 14 (SOX9) | 6 |  | 7,1033 |  |  |  |
| Treatment Day 21 (SOX9 | 6 |  |  | 9,1833 |  |  |
| Treatment Day 14 (SOX9) | 6 |  |  | 11,1183 | 11,1183 |  |
| Control Positive Day 7 (SOX9) | 6 |  |  |  | 11,2167 |  |
| Treatment Day 7 (SOX9) | 6 |  |  |  |  | 14,2200 |
| Sig. |  | ,995 | ,539 | ,073 | 1,000 | 1,000 |
| Means for groups in homogeneous subsets are displayed. | | | | | | |
| a. Uses Harmonic Mean Sample Size = 6,000. | | | | | | |
