# Supplementary material for: Osteogenic potential of gingival stromal progenitor cells cultured in platelet rich fibrin is predicted by core-binding factor subunit-α1/Sox9 expression ratio ( in vitro)
Source: F1000Res. 2018 Jul 25;7:1134. [Version 1] doi: 10.12688/f1000research.15423.1 (PMC6097418; doi:10.12688/f1000research.15423.1)
Supplement: Peer review history

# Peer review: Referee response for version 1

**DOI:** 10.5256/f1000research.16808.r36777

**Published:** 2018-08-16

**Authors:** Benny M. Soegiharto

**Affiliations:** 1 Department of Orthodontics, Faculty of Dentistry, University of Indonesia, Jakarta, Indonesia

First of all, please allow me to congratulate the authors for attempting to undertake this project which I found very interesting and of valuable additional knowledge. The manuscript itself is well-written and well-structured and I have to also commend the authors for this matter. However, I may require some clarifications on the following issues:

1.  What would be the arguments of dividing into the 3 groups as well as using such media treatment for each group (GSPC, + and - groups). This would be beneficial for readers to replicate in future use.
2. What would be the reason for determining  the evaluation of osteogenic differentiation on Day 7, 14 and 21 cell groups? At the end of that sentence, there was a quote of another work. However, I think it would make this manuscript even clearer if this could be briefly mentioned.

I have read this submission. I believe that I have an appropriate level of expertise to confirm that it is of an acceptable scientific standard, however I have significant reservations, as outlined above.

# Peer review: Referee response for version 1

**DOI:** 10.5256/f1000research.16808.r36776

**Published:** 2018-08-15

**Authors:** Ananto Ali Alhasyimi

**Affiliations:** 1 Department of Orthodontics, Faculty of Dentistry, Gadjah Mada University (UGM), Yogyakarta, Indonesia

The authors of this study have investigated the role of  GSPCs cultured in PRF in increasing the CBF-α1/Sox9 expression ratio during osteogenic differentiation. Based on the study results, the authors have concluded that the GSPCs cultured in PRF increased CBF-α1/Sox9 expression on Day 7. The results are certainly interesting and the authors are commended for executing this study. 

 However, the authors need to address the following minor concerns: 

**General**:

1. Paper is well organized and easy to follow.
2. Novelty is sufficient and high impact.
3. To improve the readability, It is recommended that the text is checked by a native English speaking person as many of the sentences might be misunderstood. I suggest a revision of the English grammar structures by an expert editor in revising manuscripts.

**Introduction**:

1. The authors have mentioned “Furthermore, a second objective was …”, however, I didn’t find the first objective. Please change this section
2. The Introduction section is too long, you can try to simplify this section (please move some of the key mechanism of the materials in enhancing CBF-α1/Sox9 expression in the discussion part

 

**Results:**

1. Please provide and add some information about standard deviation of the data in the diagrams.
2. Please indicate whether the difference is significant or not in your graph/ diagrams as well, using some symbols (asteric).

**Discussion**:

1. Discussion of the results is quite comprehensive. In analyzing the results, the authors also show citations from the previous study to support the explanation of these results.
2. The answer to the hypothesis of this study should be included at the beginning of the discussion section.
3. Please mention the limitation of this study in the discussion section for

 

**References**:

 The supporting references are too long for the medium article

I have read this submission. I believe that I have an appropriate level of expertise to confirm that it is of an acceptable scientific standard.

# Peer review: Referee response for version 1

**DOI:** 10.5256/f1000research.16808.r36488

**Published:** 2018-08-13

**Authors:** Guang Hong

**Affiliations:** 1 Liaison Center for Innovative Dentistry, Graduate School of Dentistry, Tohoku University, Sendai, Japan

This paper needs major revision. I would make the following comments.

**Introduction** **:**

 1. In final part, you said “Furthermore, a second objective was …”, however I cannot find your first objective. What is the first objective of your study?

  I think you should change this part to “The objective of this study was to analyze GSPCs cultured in PRF osteogenic differentiation predicted by CBF-α1/Sox9 expression ratio. The hypothesis of the current study is that GSPCs cultured in PRF can increase the CBF-α1/Sox9 expression ratio during osteogenic differentiation.” 

 2. The reason that why you do this research in necessary in Introduction section. And also please add the that is the problem of current and past research regarding relationship of osteogenic and CBF-α1/Sox9 ratio? 

**Methods:**

 3. The materials section is too long, you can try to simplify this section. 

**Discussion** **:**

 4. The answer of hypothesis of this study should be included in the discussion section. 

 5. The discussion about the experiment method and selection of material is necessary. There was no discussion why expression differences existed. The authors need to discuss based upon the mechanism otherwise the paper is going to be a laboratory report.  

 6. Please add the clinical implications of your study and clinical significance of the findings in discussion section. 

 7. What is the limitation of this study? You should indicate at this section. 

**Figure** **:**

 8. Fig. 1 and Fig. 3: Please indicate which pic is A, B, C……. and H, I. 

 9. Fig. 2 and Fig. 4: Please add the SD (standard deviation) to your bar graph. And if possible also add the results of statistical analysis to your graph.

I have read this submission. I believe that I have an appropriate level of expertise to confirm that it is of an acceptable scientific standard, however I have significant reservations, as outlined above.
